# Point-of-care testing of cardiovascular risk factors in Viennese community pharmacies: A cross-sectional study

**DOI:** 10.1016/j.pmedr.2025.103209

**Published:** 2025-08-10

**Authors:** Thorsten Bischof, Viktoria Mair, Alexander Schmidt-Ilsinger, Philipp Saiko, Susanne Ergott-Badawi, Bernd Jilma, Stefan Deibl, Christian Schoergenhofer

**Affiliations:** a Department of Clinical Pharmacology, Medical University of Vienna, Vienna, Austria; b Austrian Chamber of Pharmacists, Vienna, Austria

**Keywords:** Primary health care, Cardiovascular risk, Point-of-care testing, Screening, Community pharmacy

## Abstract

**Objective:**

Despite being the leading contributor to global mortality, cardiovascular diseases remain undetected in many individuals at-risk. The aim of this study was to evaluate the proportion of individuals with unrecognised cardiovascular risk factors among all-comers in community pharmacies.

**Methods:**

This cross-sectional study conducted in 47 Viennese community pharmacies between November 1, and December 31, 2024, screened adults for cardiovascular risk factors using point-of-care testing (HbA1c, lipid profile) and the age-appropriate Systematic Coronary Risk Evaluation 2. An optional follow-up after three months assessed whether medical referral occurred and pharmacotherapy was initiated or changed. Patients treated for diabetes or hyperlipidaemia were excluded.

**Results:**

Among 445 individuals, 51 % were identified with increased cardiovascular risk factors. Of these, 151 agreed to participate in the follow-up, in which 66 % were successfully contacted. The respondents reported an attendance rate of 57 % and 43 % received new or modified pharmacotherapy.

**Conclusions:**

Community pharmacy-based screening proved effective, identifying numerous previously undiagnosed individuals with increased cardiovascular risk, which is especially remarkable within a comprehensive healthcare system. However, follow-up rates remained low, indicating a gap between risk detection and medical action. A stronger integration of screening interventions with primary care and follow-up pathways with medical institutions may enhance the effectiveness.

## 1 Introduction

Cardiovascular diseases are the leading cause of death worldwide, accounting for over 20 million cases annually (Lindstrom et al., 2022). Although incidence rates have declined, the burden remains high, particularly outside high-resource groups (Conrad et al., 2024; Abdalla et al., 2020). In Austria, cardiovascular diseases accounted for 35 % of all deaths in 2023, making them the leading cause of death (Austria, 2024).

Three key modifiable risk factors – diabetes, hyperlipidaemia, and hypertension - are preventable or delayable through lifestyle modification and/or appropriate treatment (Powell-Wiley et al., 2021; Visseren et al., 2021). However, many at-risk individuals remain undiagnosed: ∼80 % of U.S. adults could benefit from a preventive intervention (Kahn et al., 2008), and ∼ 45 % of type 2 diabetes mellitus cases worldwide remain undiagnosed (Flood et al., 2021).

Community pharmacies, due to their accessibility, their high daily customer volume, and walk-in availability, provide an attractive setting for screening strategies. In many countries (e.g., United States of America, Australia, Canada, Portugal, the Netherlands) pharmacists are authorized to conduct point-of-care tests, which provide rapid and reliable measurements for key cardiovascular risk factors (INd and Ekpoh, 2023). Importantly, such point-of-care tests are affordable and therefore an interesting option for patients without a healthcare insurance.

A recent legal amendment in Austria authorized community pharmacists to perform capillary blood sampling and conduct point-of-care assays. This study aimed to evaluate the proportion of individuals with undiagnosed cardiovascular risk factors identified by point-of-care testing among all-comers in Viennese community pharmacies.

## 2 Methods

### 2.1 Study design and setting

This cross-sectional study was conducted between November 1, 2024, and December 31, 2024, in community pharmacies across Vienna, Austria. The Austrian Chamber of Pharmacists invited 340 community pharmacies to participate via written invitation. Participation required the availability of point-of-care assays for glycated haemoglobin A1 (HbA1c), total cholesterol and high-density lipoprotein. Sixty-two pharmacies agreed to participate, and 47 of these, located in 21 of Vienna's 23 municipal districts, successfully conducted cardiovascular risk screenings.

### 2.2 Study population

Eligible subjects were adults (≥18 years), who wanted to perform point-of-care tests, and met the screening recommendations of the Austrian Diabetes Society for HbA1c testing (Table S1), or the European Society of Cardiology for primary prevention of cardiovascular risk factors (Table S2). Patients already treated for diabetes or hyperlipidaemia were excluded. All participants provided written informed consent before enrolment. The independent Ethics Committee at the Medical University of Vienna (registration number: 1865/2024) and the City of Vienna (24–120) approved the study.

### 2.3 Study procedures

Participating pharmacists completed mandatory training sessions before the start of the study to standardise procedures and electronic case report form entries. Pharmacists recruited subjects by inviting all-comers. Participants were charged 10 Euros for point-of-care testing.

Cardiovascular risk was assessed based on HbA1c levels and/or the Systematic Coronary Risk Evaluation 2 (SCORE2) algorithm, including SCORE2-Older Persons (SCORE2-OP) for those aged ≥70 years (Group Sw and Collaboration ECr, 2021; *Eur. Heart J.*, 2021). This algorithm estimates the 10-year risk of first-onset cardiovascular disease based on age, sex, smoking status, systolic blood pressure, total cholesterol, and high-density lipoprotein cholesterol levels (Group Sw and Collaboration ECr, 2021; *Eur. Heart J.*, 2021). For participants younger than 40 years, the minimum eligible age of 40 was applied to enable SCORE2 risk estimation. In addition to these parameters, race was recorded, and to calculate body mass index, body weight and height were documented.

Capillary blood samples were collected regardless of fasting status. Measurements for HbA1c, total cholesterol, and high-density lipoprotein were conducted immediately after sample collection, in accordance with the manufacturers' instructions. Cartridge-based assays were exclusively employed in this study, including the Abbott Afinion 2 (Chicago, Illinois, USA), Roche cobas b101 (Basel, Switzerland), and Sysmex TSimplex TAS 101 Analyzer (Vienna, Austria), utilizing either immunoassay or enzymatic photometric detection methods. Results were typically available within 10 min. Pharmacists' measured blood pressure after a 10-min rest period. All test results and variables were entered in electronic case record forms.

Results were communicated to participants both verbally and in written form. Participants identified with increased cardiovascular risk factors were advised to consult their treating physicians or a medical institution. During informed consent, participants were asked, whether the study staff may contact them after three-to-four months for a follow-up assessment, which was conducted via telephone or email and served the purpose of assessing whether a medical appointment had been scheduled or attended and whether pharmacotherapy was initiated or adapted.

### 2.4 Outcomes

The primary endpoint was the proportion of subjects with increased cardiovascular risk factors, defined as HbA1c > 5.7 % and/or a high or very high risk according to the SCORE2(-OP), equivalent to a risk ≥2.5 % in subjects <50 years, ≥ 5 % in participants 50–69 years, and ≥ 7.5 % in individuals ≥70 years of age. Secondary endpoints included the successful referral rate in patients with elevated cardiovascular risk and the initiation or change in pharmacotherapy. A complete list of secondary endpoints is listed in the supplement (Table S3).

### 2.5 Statistical analysis

A formal sample size calculation was not performed. However, we aimed to include as many subjects as possible. Demographics and baseline characteristics, as well as primary and secondary endpoints, were summarised by descriptive statistics (absolute and relative frequencies, means and standard deviation (SD)). Group differences were explored using the Mann-Whitney-*U* test for metric variable or the Chi2 test for nominal variables, for the following groups: (i) successful follow-up vs. no successful follow-up, (ii) fulfilment of referral vs. no fulfilment of referral, (iii) new or modified pharmacotherapy vs. no changes in pharmacotherapy. Due to the exploratory nature of this analysis, we did not correct for multiple testing. Statistical analyses were performed in R-studio (Version 2023.06.0 + 421, Vienna, Austria).

## 3 Results

This study included 469 participants, of whom 445 met the eligibility criteria and provided valid measurements. Twenty-four participants were excluded due to prior diagnosis of diabetes (25 %) or missing data (75 %). The study population was predominantly white (96 %) and included more female (71 %), with a mean age of 52.4 ± 17.4. Demographics and baseline characteristics are summarised in Table 1.

**Table 1:** Demographics and baseline characteristics of the study population screened for cardiovascular risk in Viennese community pharmacies (November–December 2024).

Parameter		All subjects (*n* = 445)	Subjects with increased cardiovascular risk factors (*n* = 225)	Subjects successfully contacted in the follow-up (*n* = 99)	Subjects with initiation or change in pharmacotherapy (*n* = 24)
Age	Mean ± SD	52.4 ± 17.4	61.5 ± 16.2	62.6 ± 16.5	61.3 ± 14.8
Sex (female)	N (%)	315 (71)	126 (56)	54 (55)	11 (46)
Smoker	N (%)	77 (17)	58 (26)	26 (26)	6 (25)
Body mass index [kg/m^2^]	Mean ± SD	25.2 ± 4.6	26.2 ± 4.6	26.0 ± 3.8	25.0 ± 4.1
Race White Black/ African American Asian Other	N (%)	425 (96) 5 (1) 9 (2) 6 (1)	213 (95) 3 (1) 5 (2) 5 (2)	97 (98) 0 (0) 1 (1) 1 (1)	24 (100) 0 (0) 0 (0) 0 (0)
Systolic blood pressure [mm Hg]	Mean ± SD	130 ± 20	139 ± 21	140 ± 21	140 ± 19
Diastolic blood pressure [mm Hg]	Mean ± SD	82 ± 12	85 ± 12	85 ± 13.2	83 ± 13
Total cholesterol [mg/dL]	Mean ± SD	201 ± 41	208 ± 43	215 ± 43	215 ± 40
High-density lipoprotein [mg/dL]	Mean ± SD	64 ± 17	59 ± 16	59 ± 16	60 ± 15
HbA1c [%]	Mean ± SD	5.3 ± 0.7	5.5 ± 0.8	5.5 ± 0.8	6.0 ± 1.2
SCORE2 [%]	Mean ± SD	3.3 ± 3.3 (*n* = 368)	5.7 ± 3.8 (*n* = 150)	6.2 ± 4.4 (*n* = 63)	6.2 ± 3.1 (*n* = 18)
SCORE2-OP [%]	Mean ± SD	21.2 ± 11.1 (*n* = 77)	21.6 ± 10.8 (*n* = 75)	21.7 ± 10.4 (*n* = 36)	22.0 ± 7.3 (*n* = 6)

HbA1c: glycated haemoglobin A1c, SCORE2: Systematic Coronary Risk Evaluation 2, SCORE2-OP: Systematic Coronary Risk Evaluation 2-Older Persons, SD: standard deviation.

Elevated HbA1c levels were identified in 21 % of the study population, including 17 % with values in the pre-diabetic range (5.7–6.5 %) and 4 % with HbA1c levels consistent with diabetes (≥ 6.5 %). Elevated SCORE2 risk scores were observed in 41 % of all included subjects. Specifically, 9 % of those under 50 years of age had a SCORE2 ≥ 2.5 %, 16 % of those aged 50 to 69 years had a SCORE2 ≥ 5 %, and 17 % of the participants aged 70 years or older had a SCORE2-OP ≥7.5 %. Additionally, 16 % of all individuals had elevated blood pressure (> 140/90 mmHg) (Visseren et al., 2021).

Among the 445 participants included in this study, 51 % had increased cardiovascular risk based on HbA1c > 5.7 % and/or a high or very high risk according to the SCORE2-OP. Of these, 34 % agreed to participate in the follow-up, and among them, 66 % were successfully contacted. Of those contacted, the referral rate to a medical consultation was 57 %. As a result of these consultations, the initiation or the modification of pharmacotherapy occurred in 43 %.

In an exploratory manner, we investigated between group differences and found that smokers were less likely to fulfil the referral to physicians than non-smokers (*p* = 0.03). Otherwise, no significant group differences were found.

## 4 Discussion

This study demonstrated community pharmacy-based screening may serve as a feasible and effective approach to identify individuals with previously unknown cardiovascular risk. More than half of the participants were previously unaware of their risk, highlighting the potential for accessible entry points for preventive healthcare.

Pharmacy-based interventions have been successful in other public health areas, such as smoking cessation (Carson-Chahhoud et al., 2019), weight management (Brown et al., 2016) and management of cardiovascular risk factors (Ifeanyi Chiazor et al., 2015). Our findings reinforce their role in risk detection, even in a high-resource environment like Austria, with a comprehensive healthcare system covering almost the entire population and a region with moderate risk for cardiovascular diseases according to the European Society of Cardiology (Group Sw and Collaboration ECr, 2021; *Eur. Heart J.*, 2021). The prevalence of undiagnosed risk factors indicates gaps in the current healthcare system and underscores the value of opportunistic screening.

However, translating risk detection into medical follow-up remains a challenge. Despite personalised recommendations, only about half of the contacted at-risk individuals reported undergoing medical follow-up. This aligns with prior evidence that evaluated opportunistic screening for diabetes and cardiovascular risk in community pharmacies (Willis et al., 2014). While high rates of previously undiagnosed cases were found, a single awareness-raising effort may not be sufficient to trigger behavioural change (Willis et al., 2014). The lack of follow-up may diminish the clinical, and possibly also the economic impact of such screening interventions.

A stronger integration of community pharmacists into primary care pathways may address this gap (Hayhoe et al., 2019). Structured follow-up strategies, including medical referrals, telephone outreach, appointment reminders, and general assistance, have been shown to improve continuity of care (Krieger et al., 1999). In addition, digital health tools (i.e., mobile health applications, tele-pharmacy, communication platforms) can support patient education, reinforce medical advice, and improve adherence to recommended care, thereby increasing the overall effectiveness of pharmacy-based screening interventions (Aboelzahab et al., 2025). Future studies should assess whether proactive models, such as booking follow-up appointments directly or automated reminder systems, lead to improved outcomes.

The SCORE2(-OP) algorithm, developed by the European Society of Cardiology, provided an age-adjusted estimate of cardiovascular risk (Group Sw and Collaboration ECr, 2021). SCORE2-OP values are typically higher, reflecting the age-related increased baseline risk in the elderly (*Eur. Heart J.*, 2021). While these tools help in risk stratification, they are not designed to guide pharmacotherapy. Treatment decisions should always consider individual benefit-risk assessments, especially in older adults, in whom side effects, quality of life, and polypharmacy are critical factors. Non-pharmacological recommendations (e.g., lifestyle modifications) remain a cornerstone of cardiovascular risk reduction (Visseren et al., 2021). Notably, there is no clear indication for pharmacotherapy in individuals with pre-diabetes. Current guidelines highly recommend lifestyle changes (e.g., diet, exercise, weight management) as first-line treatment (class Ia), while pharmacotherapy may be considered (class IIa) for individuals at high cardiovascular risk based on SCORE2 (Visseren et al., 2021). This may explain the modest rate of pharmacological interventions observed during follow-up. This rate may increase over time, if lifestyle modification is not sufficient.

Several limitations should be considered. First, the testing fee may have introduced selection bias, potentially excluding individuals from lower socioeconomic backgrounds or those who perceived themselves as low risk. Participants who consented to follow-up may have been more health-conscious or motivated to act on pharmacists' advice, while those who had already consulted a physician might have been more likely to respond to follow-up attempts. Thus, the reported referral fulfilment rate may be overestimated. The study population included a higher proportion of females, who generally have a lower cardiovascular risk and higher participation rates in health checks (Group Sw and Collaboration ECr, 2021; Cook et al., 2016). In addition, participants were almost exclusively white, reflecting national demographics, which limits generalisability and precludes subgroup analyses. At the pharmacy level, acceptance of the screening service was limited. Barriers such as incompatible testing equipment, lack of infrastructure, or competing priorities may have impacted on the pharmacies' decision to participate in the study. Some participating sites did not enrol any patients, and pharmacies with underused resources (e.g., available staff, consultation space) may have been overrepresented. These factors introduce additional bias and suggest implementation challenges that may affect scalability. Consequently, the observed prevalence of undiagnosed cardiovascular risk and follow-up rates may not fully reflect those in the general population. Nonetheless, our findings support the benefits of widely accessible point-of-care testing in community pharmacies as a complementary strategy in healthcare screening programs to improve early risk detection.

## 5 Conclusions

Community pharmacy-based point-of-care testing effectively identified individuals with previously undiagnosed cardiovascular risk factors. However, its full potential may only be achieved through stronger integration with follow-up care pathways, including structured interventions that support the link between risk detection and follow-up care. Moreover, pharmacy-based screening initiatives may be especially valuable in reaching populations that experience barriers to accessing traditional healthcare services.

## CRediT authorship contribution statement

**Thorsten Bischof:** Writing – original draft, Validation, Software, Methodology, Funding acquisition, Formal analysis. **Viktoria Mair:** Writing – review & editing, Validation, Formal analysis, Data curation. **Alexander Schmidt-Ilsinger:** Writing – review & editing, Resources, Project administration, Methodology, Conceptualization. **Philipp Saiko:** Resources, Methodology, Conceptualization. **Susanne Ergott-Badawi:** Resources, Methodology, Conceptualization. **Bernd Jilma:** Writing – review & editing, Supervision, Methodology, Conceptualization. **Stefan Deibl:** Writing – review & editing, Resources, Methodology, Conceptualization. **Christian Schoergenhofer:** Writing – review & editing, Validation, Supervision, Funding acquisition.

## Funding

This study was supported by the Austrian Chamber of Pharmacists.

## Declaration of competing interest

The authors declare the following financial interests/personal relationships which may be considered as potential competing interests: Thorsten Bischof, Christian Schoergenhofer reports financial support was provided by Austrian Chamber of Pharmacists. Alexander Schmidt-Ilsinger, Philipp Saiko, Susanne Ergott-Badawi, Stefan Deibl reports a relationship with Austrian Chamber of Pharmacists that includes: employment. Christian Schoergenhofer reports a relationship with Austrian Chamber of Pharmacists that includes: consulting or advisory, funding grants, and speaking and lecture fees. If there are other authors, they declare that they have no known competing financial interests or personal relationships that could have appeared to influence the work reported in this paper.

## Data Availability

The data is available from the authors upon reasonable request.

## Appendix A Supplementary data

Supplementary material Image 1
