# Supplementary material for: Point-of-care testing of cardiovascular risk factors in Viennese community pharmacies: A cross-sectional study
Source: Prev Med Rep. 2025 Aug 10;57:103209. doi: 10.1016/j.pmedr.2025.103209 (PMC12362384; doi:10.1016/j.pmedr.2025.103209)
Supplement: Supplementary file 1 — Supplementary material [file mmc1.docx]

**Point-of-care testing of cardiovascular risk factors in Viennese community pharmacies: A cross-sectional study**

**Supplementary data**

Thorsten Bischof^1^, Viktoria Mair^1^, Alexander Schmidt-Ilsinger^2^, Philipp Saiko^2^, Susanne Ergott-Badawi^2^, Bernd Jilma^1^, Stefan Deibl^2^, Christian Schoergenhofer^1^

^1^ Department of Clinical Pharmacology, Medical University of Vienna, Austria

^2^ Austrian Chamber of Pharmacists, Vienna, Austria

# Table S1: Screening recommendations of the Austrian Diabetes Society for HbA1c testing applied to assess diabetes risk in adults included in this study

| Recommendations of the Austrian Diabetes Society for HbA1c testing |
| --- |
| Everyone with risk factors:   - BMI ≥ 25 kg/m^2^, BMI ≥ 23 for people of Asian origin - Family history of diabetes (first degree relatives) - Ethnicity with increased risk of diabetes (Asian, African and Latin-American origin) - Presence of vascular diseases - Arterial hypertension (>140/90 mm Hg or people being treated with antihypertensive medication) - HDL cholesterol < 35 mg/dL or triglycerides > 250 mg/dL - Persons with polycystic ovary syndrome - Persons with hypogonadism - Persons with physical inactivity - Persons with acanthosis nigricans - Persons with non-alcoholic fatty liver disease - Persons with chronic tobacco abuse |
| Persons with pre-diabetes; a yearly screening is recommended |
| Persons who developed gestational diabetes during pregnancy, screening every 3 years is recommended |
| Persons with human immunodeficiency viruses (HIV) |
| All persons ≥ 35 years of age |
| If values are unremarkable, a screening every 3 years is recommended, A more frequent screening schedule can be sensible, e.g. based on screening results or if there are other risk factors present |

# Table S2: Screening recommendations of the European Society of Cardiology for cardiovascular risk assessment applied to the study population

| Recommendations of the European Society of Cardiology on performing screening investigations of modifiable risk factors in primary prevention |
| --- |
| In all persons with major cardiovascular risk factors:   - Family history of premature cardiovascular disease - Familial hypercholesterolemia - Persons who smoke - Persons with arterial hypertension (treated or untreated) - Persons with diabetes - Persons with raised lipid levels - Obese persons - Persons with comorbidities that increase cardiovascular risk |
| Systematic or opportunistic cardiovascular risk assessment in the general population in men >40 years of age and women >50 years of age or postmenopausal without any risk factors, a screening every 5 years is recommended, or more frequent if risk was close to treatment threshold |

# Table S3: The complete list of primary and secondary endpoints evaluated in the study

Primary endpoint:

- Absolute and relative frequencies of participants with an increased cardiovascular risk will be calculated, which is defined as an HbA1c% > 5.7% and/or a high or very high risk according to the SCORE2-(OP), whichever is applicable (equivalent to a risk estimation of ≥ 2.5% in participants < 50 years, ≥ 5% in participants 50-69 years of age, and ≥ 7.5% in participants >70 years of age).

Secondary endpoints:

- Absolute and relative frequency of patients with an Hb1Ac of 5.7-6.4 (indicative of pre-diabetes)
- Absolute and relative frequency of patients with an Hb1Ac of ≥ 6.5 (indicative of diabetes)
- Absolute and relative frequency of patients with an Hb1Ac of ≥ 5.7 (indicative of pre-diabetes or diabetes)
- Absolute and relative frequency of patients with a high risk according to the SCORE2(-OP), whichever is applicable
- Absolute and relative frequency of patients with a very high risk according to the SCORE2(-OP), whichever is applicable
- Absolute and relative frequency of patients with a high or a very high risk according to the SCORE2(-OP), whichever is applicable
- Absolute and relative frequency of patients with a high or a very high risk according to the SCORE2(-OP), whichever is applicable, and an increased HbA1c% (≥5.7%, 5.7-6.4%, ≥6.5%)
- Number of successful referrals in patients with a high or very high risk according to the SCORE2(-OP), whichever is applicable, and/or an increased HbA1c% (≥5.7%) – if this number exceeds n= 200, then n=200 will be randomly selected and contacted. Alternatively, other pieces of evidence of contact with medical institutions in context with this screening investigation will be sought. This will be done >3 months after the initial screening.
- Absolute and relative frequency of patients with a high blood pressure (>140/90mmHg) who receive a recommendation to repeat measurements of blood pressure at home.
